# The Alexis^®^ system for laparoscopic splenectomy in pediatric patients

**DOI:** 10.1007/s13304-021-01023-5

**Published:** 2021-04-20

**Authors:** Emanuele Trovalusci, Marco Gasparella, Cristina Pizzato, Paola Midrio

**Affiliations:** 1 Pediatric Surgery, Ca’ Foncello Hospital, 31100 Treviso, Italy; 2 Pediatric Oncology Unit, Ca’ Foncello Hospital, 31100 Treviso, Italy; 3 Pediatric Surgery, University of Padua, 35128 Padua, Italy

**Keywords:** Splenectomy, Alexis, Laparoscopic, Pediatric, Mini-invasive surgery

## Abstract

The laparoscopic splenectomy in pediatric patients is performed worldwide but often the disproportion between size of patients and size of organs requires an extra laparotomic access for spleen removal. The aim of the present study was to evaluate the safety and effectiveness of the Alexis^®^ system to retrieve the spleen without additional laparotomic access. The charts of all patients who underwent splenectomy at our center during the last 5 years were retrieved. In all the cases the Alexis^®^ system was placed in the umbilicus, thru which a 10 mm camera was inserted. Three additional 5 mm standard trocars were inserted. Seven patients, affected by spherocytosis (3), epidermoid cyst (2), idiopathic thrombocytopenic purpura (2) and thalassemia (1), underwent laparoscopic splenectomy at a median age of 10 years (range: 8–17). Median patients’ weight was 32.5 kg (range: 25–71) and spleen size 15 cm (11–18). In all the cases, upon removal of the camera, the retrieval bag was inserted thru the umbilicus under direct view, the spleen retrieved, morcellated, and removed. No conversion nor enlargement of one of the ports nor an extra laparotomic access were required. The patients were discharged on the fifth post-operative day and the cosmetic results were excellent. Removal of the spleen can be safely performed without any additional laparotomy thru the Alexis^®^ system placed in the umbilicus. This system is effective also in case of major patient/organ size disproportion and the final cosmetic aspect is excellent.

## Introduction

Splenectomy is usually performed as treatment of hematologic diseases, such as hereditary spherocytosis (HS), idiopathic thrombocytopenic purpura (ITP) or sickle cell disease (SCD), to slow down the blood cells turnover [1]. If possible, splenectomy should be delayed until the patient is 4–6 years old because, according to the literature, this reduces the incidence and mortality of overwhelming post-splenectomy infections, as well as routine immunization against Streptococcus Pneumoniae, Neisseria Meningitidis and Hemophilus Influenzae type B and antibiotic prophylaxis for 2–5 years. [1, 2]

A breakthrough in the management of these patients was the advent of the laparoscopic splenectomy, performed for the first time in 1991 by general surgeon Delaitre [3] and, only 2 years after, by Tulman in the pediatric population [4].

Despite the attractiveness this technique gained over time among general and pediatric surgeons, some authors were skeptical about the real benefits compared to the open approach. The main criticisms were centered on the higher costs due to prolonged operative time, the lower capacity of detecting accessory spleens, and the technical difficulties to remove the organ, especially in those small patients with large spleens [5, 6].

The development of smaller laparoscopic instruments and the improvement of laparoscopic skill in pediatric age, equal rates of accessory spleens detection, shorter patients’ hospitalization that compensates the higher operative costs of laparoscopic, better esthetic results, pain reduction, and less post-operative complications (cit) have been reported [7–9]. An open issue remains the extraction of voluminous organs from small patients that require either an extra laparotomic access or the enlargement of one of the trocar site [10, 11].

Aim of this study is to demonstrate that the use of the Alexis^®^ wound retractor system can further improve this technique overcoming the problem of large spleen retrieval and better cosmetic outcome for the patient.

## Material and methods

All patients who underwent splenectomy during the last 5 years at the Pediatric Surgery of Ca’ Foncello Hospital of Treviso (Italy), were included in the present analysis and their medical records retrieved from the archive. For each case, diagnosis, clinical data, pre-operative imaging and therapy, and post-operative course were analyzed.

IRB approval was not required as we described an innovative variation on existing technique and all patients’ data has been anonymized.

### Surgical technique

The surgical technique starts with a 2 cm umbilical incision to insert a Small size Alexis^®^ wound retractor system—with its laparoscopic cap and a Kii Fios First Entry^®^ bladeless 12 mm trocar—thru which a 10 mm camera is placed. Then, other three additional 5 mm standard trocars are inserted, one in the epigastric region and two along the midline above and below the umbilicus. After selective hilar vessel division with diathermic LigaSure™, a 5 mm laparoscopic optic is placed in one of the operative trocars and a 15 cm retrieval bag advanced thru the Alexis^®^ upon removal of the cap and the trocar. After repositioning the cap and reestablishing an adequate pneumoperitoneum, the specimen is inserted into the bag and moved under the umbilical access. At this point, the Alexis^®^ cup is removed again and the bag opened thru the ring so then the surgeon can proceed with the manual and instrumental morcellation of the spleen under direct view. The last step is the extraction of the retrieval bag from the wound dilatator without necessity to enlarge the incision, then the surgeon can proceed with the hemostasis and closure of the surgical accesses.

## Results

From 2015 to 2019, a total of 7 children, 4 males and 3 females, underwent laparoscopic splenectomy in our center. The median age of patients at the time of surgery was 10 years (range: 8–17 years.) and their median weight 32.5 kg (range: 25–71 kg). The majority was affected by hematologic diseases—3 cases of hereditary spherocytosis, 1 of idiopathic thrombocytopenic purpura and 1 of beta-thalassemia—while 2 patients were diagnosed of epidermoid cyst.

Pre-operative imaging was performed in all the patients: hematologic ones were periodically followed up with ultrasonography, while epidermoid cysts imaging was accomplished using magnetic nuclear resonance. Median longitudinal diameter of the spleen was 15 cm (range: 11–18 cm).

Prophylactic vaccinations were administered to all patients before surgery.

Total splenectomy was performed in 6 patients, while partial splenectomy was resolutive for 1 patient with epidermoid cyst. No complications nor need to convert occurred during the procedure. Only one patient needed an intraoperative blood transfusion of 300 ml. In none of the cases, the enlargement of a port or an extra laparotomic access were required for morcellation or retrieval bag extraction. An accessory spleen was detected intraoperatively in the patient with epidermoid cyst who underwent total splenectomy, and for this reason it was not removed. Mean operative time was 205 min (range: 133–299 min), including time to morcellate the spleen. Two patients with HS had a concomitant cholecystectomy for biliary sludge and clinical history of cholelithiasis; hence, an additional 5 mm trocar was positioned in the right hypochondrium. Moreover, 1 of them underwent incidental appendicectomy.

Post-operative course was regular in 6 patients, with discharge on the 5th post-operative day (range: 4–7 days). Per internal protocol, a Doppler echocardiography of abdominal vessels was performed 1 week after surgery and that was normal in all of the cases.

One patient required embolization of a pancreatic vessel on the 4th post-operative day for persisting bleeding, and was discharged 14 days after.

Five patients begun daily amoxicillin prophylaxis to be maintained until 18 years of age.

Main patient characteristics are reported in Table 1.

**Table 1:** Patient main characteristics

Patients	Age (years)	Weight	Diagnosis	Spleen size (cm)	Other procedures	Operative time (min)	Trasfusions (ml)	Accessory spleens	Post-operative complicances
1 ♂	8	26	Epidermoid cyst (8 cm)	11	No	205	–	No	No
2 ♂	17	71	ITP	13.6	No	133	–	No	Pancreatic branch leakage
3 ♂	10	27.1	HS	18	Cholecystectomy + appendicectomy	289	–	No	No
4 ♀	10	25	HS	17	No	181	300	No	No
5 ♀	14	52.9	HS	17.5	cholecystectomy	299	–	No	No
6 ♀	8	32.5	β-thalassemia	14.5	No	145	–	No	No
7 ♂	11	35	Epidermoid cyst (14 cm)	15	No	250	–	One, 22 mm (not removed)	No

## Discussion

Laparoscopic splenectomy has begun the gold standard in both pediatric and adult population with better cosmetic results, shorter hospitalization, and quicker return to daily activities [12].

One of the most controversial aspect of splenectomy, especially among pediatric surgeons, is the suitability of laparoscopic approach in presence of splenomegaly, considering the reduced intrabdominal space. Data showed that spleen weight > 500 g is associated to higher operative time and rate of conversion [7] and some authors suggest to not perform laparoscopic splenectomy in case of splenomegaly [10]. The cut-off for splenomegaly in children still remains unclear. The value for adults has been applied (> 15 cm, massive spleen > 20 cm), but this datum is useless if not related to the size and age of the patient [13]. The European Association for Endoscopic Surgery, for example, defines massive splenomegaly in children a spleen larger than four times the normal size for age [14].

In presence of radiologically documented splenomegaly, some surgeons discourage laparoscopic splenectomy in favor to open surgery [15]. Others suggest a peri-operative splenic artery embolization to reduce the organ size and ease the laparoscopic splenectomy [16]. Another technique is the Hand Assisted Laparoscopic Splenectomy (HALS), which combines the laparoscopic and the open surgery approaches thanks to the introduction of an assistant’s hand in the abdominal cavity thru an enlarged laparoscopic access or a Pfannenstiel incision.

Despite the increased popularity of laparoscopic splenectomy among pediatric surgeons, there is neither a standardized procedure nor a consensus about some technical aspects, considering also the low amount of cases per year [14, 17]. Patient position is variable according to surgeon choices: the anterior approach with patient supine allows a better visualization and control of the splenic hilum, and the possibility to right tilt the table to facilitate the splenic isolation during last steps of splenectomy. On the other hand, the posterolateral approach, with patient laying on his right side, makes easier the ligaments dissection and spleen isolation without the use of retractors because spleen and abdominal organs shift by gravity [18]; moreover, the better visualization of pancreatic tail reduces the risk of injuries [14]. However, the posterolateral approach is not indicated when other procedures are requested, such as cholecystectomy. Different approaches are described even for vascular isolation, which can be performed by individual vessel or en-bloc dissection [19]. In our center, the anterior approach is preferred, with the patient supine and the splenic hilum approached by individual vessel isolation and dissection with LigaSure™.

Spleen retrieval is the last challenging step of laparoscopic splenectomy, especially in case of big spleen and small size children. According to an Italian multicentric survey, the spleen extraction is equally performed thru an accessory Pfannenstiel incision or by means of an endo-bag and enlargement of a port’s size [17]. Other authors suggest their personal technique to retrieve as fast as possible the specimen [20], but there is no agreement on the maximum spleen volume which allows to use endo-bags in an efficient and time-saving way. In all of our cases, even in one patient with a spleen longitudinal diameter of 18 cm, sample retrieving was performed without complications using an endo-bag inserted thru the Alexis^®^ system, with no need of accessory laparotomy nor enlargement of a trocar site.

**Fig. 1:**
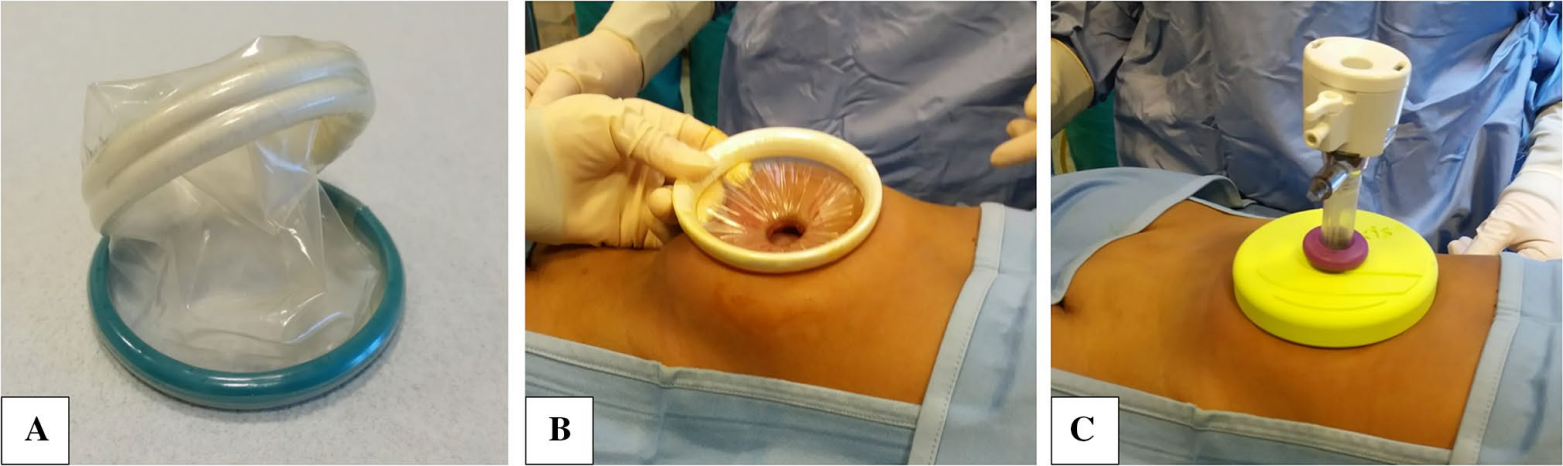
**a** Alexis^®^ wound retractor system; **b** device placed in the umbilical access, with wound under tension; **c** after cap placement, which allows to insert a 12 mm trocar and establish pneumoperitoneum, converting the device into a laparoscopic access

The Alexis^®^ wound retractor system is a device consisting of a flexible and transparent cylinder-shaped membrane with 2 semirigid rings at both the extremities. After placing one ring inside the surgical wound, the surgeon proceeds rolling the external one to fold the membrane on itself until it reaches the adequate tension and adherence to the wound. In this way, the device circumferentially enlarges the surgical incision, allowing a better visualization of the intrabdominal cavity and minimizing tissue trauma (Fig. 1). The Small size Alexis^®^, indeed, has the advantage of enlarging the umbilical incision up to 6 cm without further incisions, so it is possible to quickly retrieve big size morcelled spleen obtaining a perceived scarless umbilicus. This device can also be used during laparoscopic procedures thanks to a cap which covers the external ring and converts the surgical access to a standard laparoscopic port maintaining the pneumoperitoneum; the cap also allows the insertion of any size of trocar.

The use of Alexis^®^ for splenectomy, as performed by our center, is almost comparable to the single-incision laparoscopic splenectomy (SILS) in terms of cosmetic outcomes. This procedure was developed to perform the entire splenectomy with only an umbilical incision thanks to the use of particular port systems which act as wound retractors and allow to insert up to 3 instruments thru the same access. SILS procedure has surely cosmetic advantages but also technical issues, such as the absence of triangulation and reduced instruments maneuverability, so it is performed only in few centers [21, 22]. Our technique, on the contrary, implies the use of extra 5 mm trocars which results in imperceptible abdominal scars and avoids instruments collision. Moreover, the Alexis^®^ wound retractor is less expensive than SILS devices.

In conclusion, laparoscopic splenectomies performed in our center with the Alexis^®^ have become safer and quicker than the standard laparoscopic approach, further improving this technique. In fact, there is neither necessity to enlarge the surgical incision nor create an extra laparotomic access to retrieve big specimens. Besides, the umbilical opening obtained with the Alexis^®^ allows to perform the morcellation procedure with the use of both fingers and instruments, and always under direct view, making the procedure quicker and easier and reducing the risk of accidental retrieval bag rupture with specimen spillage into the peritoneal cavity.

## Data Availability

All data generated or analyzed during this study are included in this published article.
